# Is Proteolytic Cleavage Essential for the Enhanced Activity of *Hydra* Pore-Forming Toxin, HALT-4?

**DOI:** 10.3390/toxins15060396

**Published:** 2023-06-13

**Authors:** Wei Yuen Yap, Jung Shan Hwang

**Affiliations:** 1 Department of Biological Sciences, School of Medical and Life Sciences, Sunway University, Bandar Sunway 47500, Malaysia; 18044610@imail.sunway.edu.my; 2 Department of Medical Sciences, School of Medical and Life Sciences, Sunway University, Bandar Sunway 47500, Malaysia

**Keywords:** *Hydra* actinoporin-like toxin, pore-forming toxin, dibasic cleavage site, regulated secretory pathway, cytolytic activity

## Abstract

*Hydra* actinoporin-like toxin 4 (HALT-4) differs from other actinoporins due to its N-terminal propart that contains approximately 103 additional residues. Within this region, we identified five dibasic residues and assumed that, when cleaved, they could potentially exhibit HALT-4′s cytolytic activity. We created five truncated versions of HALT-4 (tKK1, tKK2, tRK3, tKK4 and tKK5) to investigate the role of the N-terminal region and potential cleavage sites on the cytolytic activity of HALT-4. However, our results demonstrated that the propart-containing HALT-4 (proHALT-4), as well as the truncated versions tKK1 and tKK2, exhibited similar cytolytic activity against HeLa cells. In contrast, tRK3, tKK4 and tKK5 failed to kill HeLa cells, indicating that cleavage at the KK1 or KK2 sites did not enhance cytolytic activity but may instead facilitate the sorting of tKK1 and tKK2 to the regulated secretory pathway for eventual deposition in nematocysts. Moreover, RK3, KK4 and KK5 were unlikely to serve as proteolytic cleavage sites, as the amino acids between KK2 and RK3 are also crucial for pore formation.

## 1. Introduction

**Figure 1:**
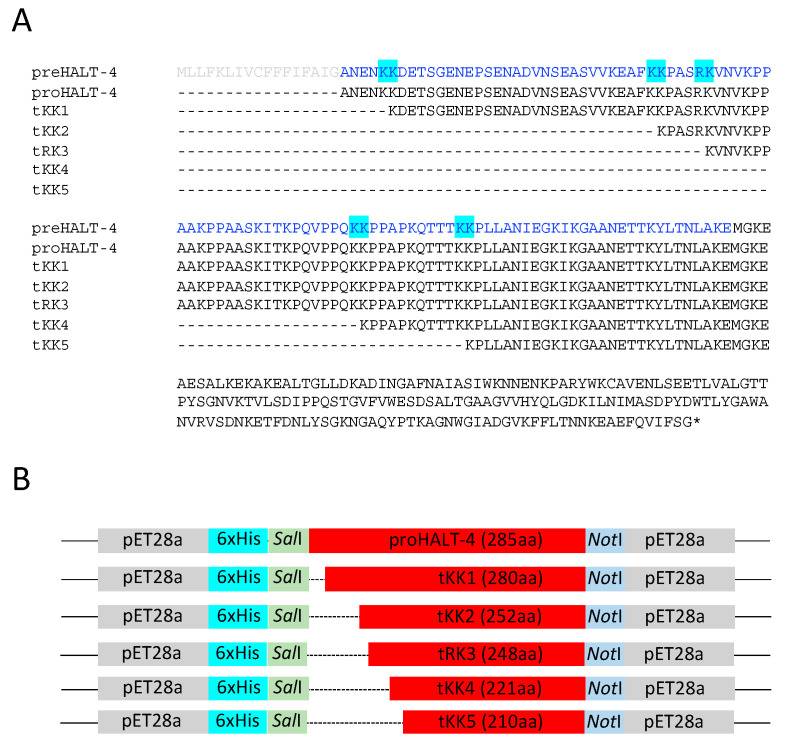
(**A**) The N-terminal amino acid sequences of HALT-4 precursor (preHALT-4), propart-containing HALT4 (proHALT-4) and five truncated HALT-4 (tKK1, tKK2, tRK3, tKK4 and tKK5) are aligned. The propart, proposed in this study, is shown in blue, the signal peptide is shown in light grey, and the five pairs of basic residues are highlighted in cyan. (**B**) A schematic drawing illustrates the orientation of recombinant proHALT-4 and truncated HALT-4 in pET28a. Dotted lines indicate the removal of amino acids in HALT-4. The number of amino acids for proHALT-4 and each truncated HALT-4 is indicated in parentheses.

*Hydra* actinoporin-like toxin (HALT) is a family of seven α-pore-forming toxins, namely HALT-1 to HALT-7 [1,2]. They were initially identified in *Hydra magnipapillata*, a freshwater hydrozoan [1]. While HALTs belong to the actinoporin family, most actinoporins are predominantly found in Anthozoa, a subphylum of Cnidaria that includes sea anemones and corals. In addition to Anthozoa, other subphyla comprise Medusozoa (subclasses hydrozoans, cubozoans, medusozoans, scyphozoans, and staurozoans) and Myxozoa. All cnidarians are characterized by specialized cell types known as nematocytes, which can secrete various toxins for both offense and defense. HALTs are a notable exception as they are the only actinoporins found outside of anthozoans. The secondary and tertiary structures of HALTs closely resemble those of actinoporins from sea anemones, particularly HALT-1, -5, -6 and -7. However, HALT-3 and HALT-4 deviate from actinoporins by having a shorter and an extended N-terminal peptide, respectively [2]. Actinoporins are stored as a reservoir in nematocysts, which are capsular organelles with long coiled threads, and are believed to play an offensive role in hunting prey. Similarly, HALT-1, -4 and -7 have also been detected in nematocyst-containing cells, while HALT-1, -2 and -6 have been detected in other cell types [2]. All HALTs, except HALT-3, contain a signal peptide at the N-terminal end. The signal peptide is meant to be cleaved during HALT protein processing in the rough endoplasmic reticulum prior to HALTs becoming mature proteins. According to Glasser et al. (2014), HALT-4 and HALT-2 contain a putative propart because of the extended N-terminal peptide, while other HALTs have zero to three residues between the signal peptide and the mature polypeptide [1]. Compared to mature HALT-1, the propart-containing HALT-4 (proHALT-4) has an additional 103 amino acids, and this region contains five paired basic sites (KK or KR) [3,4]. Therefore, HALT-4 is distinct among other HALTs as it contains a propart-like sequence that might undergo cleavage for protein maturation. The five potential cleavage sites in the N-terminal region of HALT-4 are shown in Figure 1A. A paired basic cleavage site is not found in the propart of HALT-2.

Numerous cnidarian toxins, including equinatoxin II, possess a signal peptide followed by 9–17 residues of a propart at the N-terminus. Anderluh et al. (2000) demonstrated that several genes encoding nematocyst-associated proteins and toxins have a highly conserved signal peptide and propart that ends with KR basic residues [5]. For instance, residues 1–19 of equinatoxin II correspond to the signal peptide, which directs the precursor polypeptide to the endoplasmic reticulum, while residues 20–35 represent the propart with KR as the last two residues [6,7]. Furthermore, a homolog of prohormone convertase 3 (PC3) found in vertebrates has been discovered in *Hydra*. PC3 is a calcium-dependent protease that activates precursor proteins by cleaving pairs of basic amino acids. The *Hydra* PC3-like protein shares 55.4% and 56.7% identity in the catalytic domain with mouse PC3 and human furin, respectively [8]. This finding suggests that the *Hydra* PC3-like protein may process the precursor form of HALT-4 by targeting and cleaving one or more paired basic sites at the N-terminus of HALT-4. Although recombinant proHALT-4 was capable of killing human cells, its activity was seven-fold weaker than that of recombinant EqtII [1,2]. Therefore, our study explored the possibility that HALT-4 could be enzymatically cleaved at one of the paired basic sites, resulting in a significantly enhanced cytotoxicity of the cleaved HALT-4.

## 2. Results and Discussion

### 2.1. Construction of Truncated rHALT-4

**Figure 2:**
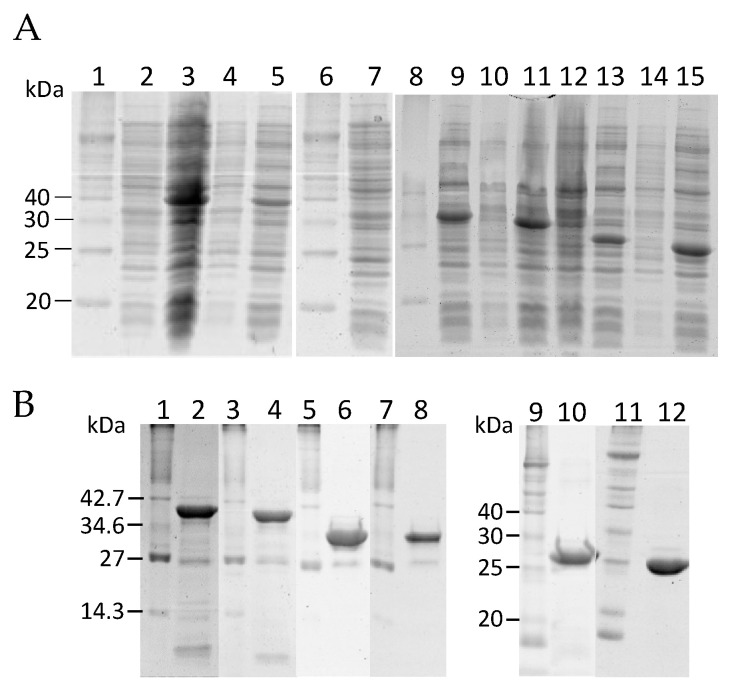
(**A**) The expression of proHALT-4 and different truncated HALT-4 in the presence of 1 mM IPTG and 3% ethanol was analyzed using 12% SDS-PAGE. Lanes 1, 6 and 8, protein markers; lane 2, proHALT-4 without IPTG; lane 3, proHALT-4 with IPTG (35.7 kDa); lane 4, tKK1 without IPTG; lane 5, tKK1 with IPTG (34.75 kDa); lane 7, tKK2 without IPTG; lane 9, tKK2 with IPTG (31.76 kDa); lane 10, tRK3 without IPTG; lane 11, tRK3 with IPTG (31.22 kDa); lane 12, tKK4 without IPTG; lane 13, tKK4 with IPTG (28.52 kDa); lane 14, tKK5 without IPTG; lane 15, tKK5 with IPTG (27.34 kDa). (**B**) The 12% SDS-PAGE analysis of proHALT-4 and different truncated HALT-4 after nickel affinity purification and dialysis. Lanes 1, 3, 5, 7, 9 and 11, protein markers; lane 2, proHALT-4; lane 4, tKK1, lane 6, tKK2; lane 8, tRK3; lane 10, tKK4; lane 12, tKK5.

Assuming that each of the five pairs of basic sites could potentially be cleaved to generate HALT-4 with higher cytolytic activity, we used proHALT-4 as a template to construct five truncated HALT-4, which we named tKK1, tKK2, tRK3, tKK4 and tKK5 (Figure 1B). The propart region (marked in blue in Figure 1A) has a higher percentage of hydrophobic residues (39.13%) compared to hydrophilic residues (33.04%). Acidic residues, including aspartic acid and glutamate, are exclusively present at both the N- and C-terminal ends of the propart. The central region primarily consists of hydrophobic residues such as proline, alanine and valine, along with the basic residue lysine. Additionally, the propart does not exhibit a specific α-helix or β-strand structure but instead possesses a coiled-coil domain. Each truncated HALT-4, with a specific paired basic site removed, was cloned into the pET28a vector, which has a 6xHis tag located at the N-terminal end of the insert. After confirming the sequence of each truncated HALT-4, along with the proHALT-4, they were transformed into Rosetta Gami 2 (DE3) *E. coli*. The recombinant proHALT-4, tKK1, tKK2, tRK3, tKK4 and tKK5 were then expressed in the presence of 1 mM IPTG and 3% ethanol, as shown in Figure 2A. The result showed the expression of the recombinant proteins, with the expected molecular weights of proHALT-4, tKK1, tKK2, tRK3, tKK4 and tKK5 being 34.75 kDa, 31.76 kDa, 31.22 kDa, 28.52 kDa, 27.34 kDa and 23.9 kDa, respectively. These recombinant proteins were then purified under denaturing conditions, using 8 M urea in the cell-disruption buffer and the binding buffer of Ni-NTA resins. During the washing steps, the urea concentration was gradually decreased (8 M, 6 M, 4 M, 2 M, 1 M and 0 M) to facilitate protein refolding within the Ni-NTA column. Finally, the protein was eluted from the column using a high imidazole concentration. The eluted protein fractions were pooled and dialyzed before being stored at −80 °C. Figure 2B shows the recombinant proHALT-4, tKK1, tKK2, tRK3, tKK4 and tKK5 after denaturing purification and dialysis.

### 2.2. Cytotoxicity of Truncated rHALT-4

To evaluate the cytolytic activity of the truncated forms of HALTs-4 (tKK1, tKK2, tRK3, tKK4 or tKK5), cytotoxicity assays were conducted on HeLa cells. Various concentrations (5 µg/mL, 10 µg/mL, 15 µg/mL, 20 µg/mL, 25 µg/mL and 30 µg/mL) of proHALT-4, tKK1, tKK2, tRK3, tKK4 and tKK5 were applied to the cells, and their cytolytic activity was assessed. Since proHALT-4 exhibited cytolytic activity, it was hypothesized that proteolytic cleavage at the paired basic site of HALT-4 is essential for enhanced activity in HeLa cells. The cytolytic activity was determined by measuring the concentration of recombinant protein that reduced cell viability by 50% (CC50). The standard deviation was taken into account for the analysis.

**Figure 3:**
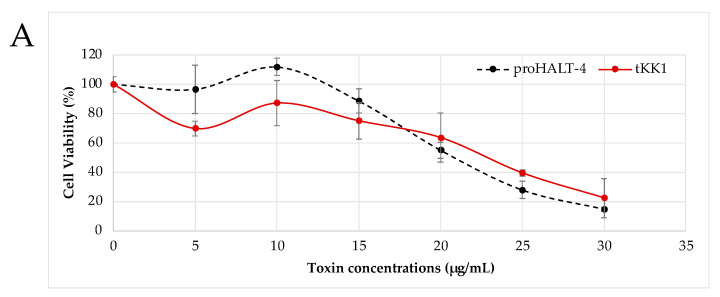

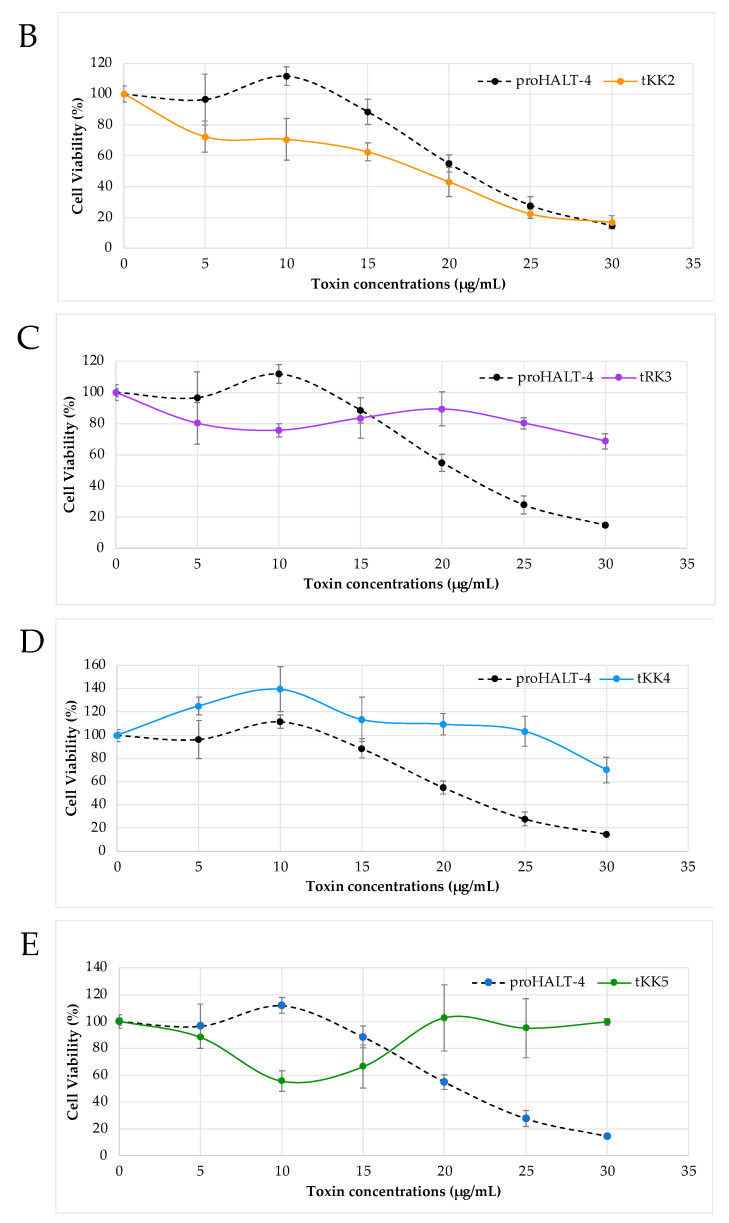
The viability of HeLa cells was assessed after treatment with various concentrations of proHALT-4 and truncated HALT-4. HeLa cells were incubated with each recombinant protein at concentrations of 5, 10, 15, 20, 25 and 30 µg/mL at 37 °C for 24 h. Cell viability was quantified at 570 nm/630 nm, and each experiment was conducted in triplicate. The results are presented as mean values accompanied by standard deviations. The comparisons were made between proHALT-4 and respective truncated HALTs: (**A**) KK1, (**B**) tKK2, (**C**) tRK3, (**D**) tKK4 and (**E**) KK5. The positive control consisted of cells treated with 100% DMSO, while the negative control consisted of untreated cells.

The results showed that tKK1 displayed no significant difference in cell viability compared to proHALT-4 (Figure 3A). Both proHALT-4 and tKK1 had similar CC50 values of 21 µg/mL and 23 µg/mL, respectively, suggesting that the removal of five residues in tKK1 had a negligible impact on its cytolytic activity. In contrast to tKK1, the N-terminus of tKK2 undergoes a more significant alteration, with the removal of 33 residues. However, its cytolytic activity remained largely unaffected. In fact, tKK2 demonstrated slightly higher cytotoxicity than proHALT-4, particularly at concentrations between 5 and 20 µg/mL (Figure 3B). Although the KK cleavage in the N-terminus of tKK2 might contribute to its enhanced cytolytic activity, the difference was not statistically significant, indicating the need for further investigation. On the other hand, tRK3, tKK4 and tKK5 did not exhibit any cytolytic activity (Figure 3C–E) (Supplementary Table S1). These results confirmed the previous observation that proHALT-4 possesses cytolytic activity [2]. The similar cytotoxic level of tKK1 and tKK2 compared to proHALT-4 suggested that the N-terminal short peptides (the first 33 amino acids of the N-terminal proHALT-4) may not play significant roles in the structure and function of HALT-4. However, it cannot be ruled out that proteolytic cleavage at the KK1 and KK2 sites facilitated the sorting of tKK1 and tKK2, respectively, to the nematocyst capsule via the regulated secretory pathway [5]. A pair of basic residues are known to act as sorting signals for targeting secretory proteins to the regulated secretory pathway, which then releases the protein to its final subcellular location [9,10]. For example, a study by Brakch et al. (1994) showed that a single mutation of the Arg–Lys pair prevented prosomatostatin from entering the regulated secretory pathway [11]. The fact that tRK3, tKK4 and tKK5 did not affect cell viability in the MTT assays (Figure 3C–E) suggests that, except for the first 33 amino acids, the remaining propart of HALT-4 may play a crucial role is cytolysis.

It is known that the six recombinant proteins (full-length and truncated forms of HALT-4) contain an additional 40 residues originating from the pET28a vector, including a His-tag and a thrombin cleavage site. In our previous study, we observed significant cytolytic activity of recombinant HALT-4 despite the presence of additional residues from the pET28a vector [2]. However, it has been reported that the presence of fusion His-tag and vector residues at the N-terminal end led to reduced activity in either hemolysis or cytolysis for several actinoporins, including magnificalysin III from *Heteractis magnifica* and sticholysins I and II from *Stichodactyla helianthus* [12]. On the other hand, recombinant AvtI hemolytic toxin from *Actineria illosa* showed a 9.2-fold increase in its hemolytic activity when expressed in *E. coli* BL21 (DE3) as compared to the native AvtI [13]. In both cases, the recombinant proteins retained their lytic activities. While the His-tag and vector residues could potentially affect the cytolytic activity of other recombinant proteins, in the case of HALT-4 (full length or truncated), they do not appear to have a substantial impact on the cytolytic activity, as shown by our previous findings. It is worth noting that even after thrombin cleavage, 23 residues of the pET28a vector still remain at the N-terminus of proHALT-4 or the truncated HALTs-4.

The N-terminal region of actinoporins plays a critical role in initiating pore formation by inserting the N-terminal domain into the membrane [14]. Thus, it typically contains an amphipathic helix. Additionally, pore formation by sticholysin, a typical actinoporin found in the sea anemone *Stichodactyla helianthus*, has been shown to be dependent on the thickness of lipid bilayer [15]. According to the findings in the study, the bilayer thickness of di-18:1 phosphatidylcholine provided the optimal conditions for the penetration of the N-terminal region of sticholysin (30 residues), which subsequently led to pore formation [15]. As the N-terminal peptide of HALT-4 lacks a distinct amphipathic region and is nearly five times longer than the N-terminal region of sticholysin, it may not be capable of membrane insertion like other actinoporins. Consequently, an intriguing area for future investigation would be to determine the role of the HALT-4 N-terminus in pore formation by determining the crystal structure of the HALT-4-lipid membrane complex.

## 3. Conclusions

Based on the present results, it can be concluded that RK3, KK4 and KK5 are not the proteolytic cleavage sites for HALT-4. Thus, the N-terminal region of HALT-4 is assumed to be longer than that of other HALTs and actinoporins. Regarding KK1 and KK2, cleavage at these sites did not increase the cytolytic activity of truncated HALT-4, and they may instead play a role in the regulated secretory pathway.

## 4. Materials and Methods

### 4.1. Construction of KK1, KK2, RK3, KK4 and KK5

Polymerase chain reaction (PCR) was performed to amplify truncated HALTs-4, KK1, KK2, RK3, KK4 and KK5. In brief, a mixture of 40 µL of ddH_2_O, 5 µL of 10× Thermopol buffer, 1 µL of 10 mM dNTPs, 1 µL of 10 µM forward primer, 1 µL of 10 µM reverse primer, 1 µL of template (pET28a inserted with *HALT*-4) [2] and 1 µL of 5 units/µL *Taq* polymerase were incubated in the thermal cycler. The following cycles were used: 94 °C for 5 min; followed by 30 cycles of 94 °C for 30 s, 55 °C for 30 s and 72 °C for 1 min; and a final extension at 72 °C for 5 min. The primers used in this experiment are listed in Table 1.

To prepare for ligation, both the PCR product and pET28a were digested with *Sal*I (20 units/µL) and *Not*I (20 units/µL) at 37 °C for 2 h. The digested DNA fragments were purified using gel electrophoresis and the QIAquick Gel Extraction Kit (Qiagen, Santa Clarita, CA, USA). Truncated *HALT*-4 was ligated to pET28a using T4 DNA ligase at 16 °C overnight. The mixture was transformed into DH5α (Invitrogen, Carlsbad, CA, USA) after ligation. Plasmid was extracted from DH5α and sent for sequencing to confirm the reading frame of truncated-*HALT*-4 in pET28a. After the sequence was confirmed, the plasmid was transformed into Rosetta Gami 2 (DE3) (Novagen, Madison, WI, USA).

### 4.2. Expression and Purification of KK1, KK2, RK3, KK4 and KK5

The expression and purification of truncated HALT-4 were carried out as described by Yap et al. [2]. In brief, an overnight culture was grown in LB broth with 50 μg/mL of kanamycin until the cell density reached OD_600_ 0.5 to 0.6. The expression of truncated-HALT-4 was then induced with 1 mM IPTG and 3% ethanol for 3 h at 37 °C with agitation. The cell pellet was collected by centrifugation and stored at −20 °C. To purify the truncated HALT-4, the cell pellet was resuspended in denaturing lysis buffer (60 mM NaH_2_PO_4_, 500 mM NaCl, 10 mM imidazole, 20 mM β-mercaptoethanol and 8 M urea, pH 8.0). Soluble cell lysate was obtained by sonication and subjected to affinity chromatography of nickel-chelating resin, Ni-NTA Superflow (Qiagen, Santa Clarita, CA, USA). The purified protein was dialyzed in a series of refolding buffers (60 mM NaH_2_PO_4_; 500 mM NaCl; 20 mM imidazole; 20 mM β-mercaptoethanol) containing decreasing concentrations of urea (8, 6, 4, 2, 1 and 0 M) and finally in 1× phosphate-buffered saline (PBS) for protein refolding. The refolded proteins were examined using 12.5% SDS-PAGE.

### 4.3. Cytotoxicity Assay

The HeLa cell line (ATCC CCL-2) was obtained from ATCC, Manassas, VA, USA. To culture HeLa cells, the thawed cells were quickly transferred into 9 mL of minimum essential medium (MEM) and centrifuged at 200× *g* for 5 min at 4 °C. The supernatant was then removed, and the cells were resuspended in 1 mL MEM. The resulting mixture was transferred to a T75 flask containing 9 mL of 10% FBS–MEM and incubated at 37 °C with 5% CO_2_.

To assess cell viability, 1 × 10^4^ HeLa cells were seeded in 10% FBS–MEM in a 96-well plate and incubated for 16 h. After removing the FBS–MEM, the cells were treated with different concentrations of truncated HALTs-4 (5 µg/mL, 10 µg/mL, 15 µg/mL, 20 µg/mL, 25 µg/mL and 30 µg/mL, which were dissolved in MEM) for 24 h. The cells were then incubated with 30 µL of 5 mg/mL 3-(4,5-dimethylthiazol-2-yl)-2,5-diphenyltetrazolium bromide (MTT) for 3 h at 37 °C. Next, the supernatant was removed and 200 µL of 100% DMSO was added into each well. The absorbance readings were measured at 570 nm with reference to 630 nm using a microplate reader (FLUOstar Omega Microplate Reader, BMG Labtech, Ortenberg, Germany). HeLa cells treated with 100% DMSO served as the positive control, while the negative control involved treating HeLa cells with 1× PBS instead of truncated HALTs-4. The assays were performed in triplicate, and the percentage of cell viability was calculated with standard deviation.

## Figures and Tables

**Table 1:** Forward and reverse primers for constructing truncated HALTs-4.

Primer	Nucleotide Sequence ^a^
KK1HALT4_FW	5′-CAG**GTCGAC**AAAAAGACGAAACCTCAGGT-3′
KK2HALT4_FW	5′-CGC**GTCGAC**AAAAACCTGCATCACGAAAA-3′
RK3HALT4_FW	5′-CGC**GTCGAC**GAAAAGTCAATGTAAAACCC-3′
KK4HALT4_FW	5′-CGC**GTCGAC**AAAAACCACCCGCACCAAAA-3′
KK5HALT4_FW	5′-GGC**GTCGAC**AAAAACCTTTGTTAGCTAAT-3′
HALT4_RV	5′-GTT**GCGGCCGC**TTACCCAGAAAAAATTAC-3′

^a^ *Sal*I (GTCGAC) and *Not*I (GCGGCCGC) were incorporated in the forward and reverse primers, respectively. They are marked in bold type.

## Data Availability

Data are contained within the supplementary material. The data presented in this study are available in Tables S1–S3.

## Supplementary Materials

The following supporting information can be downloaded at: https://www.mdpi.com/article/10.3390/toxins15060396/s1, Table S1: Raw data and average of MTT readings for proHALT-4 and truncated HALTs-4; Table S2: Controls for MTT assays; Table S3: Standard deviation of MTT assay.
